# TRAIL mediated apoptosis ruling and anticancer trigger by fine-tuned nano spheres of *Fagonia cretica* methanolic extracts as novel cancer regime

**DOI:** 10.1038/s41598-023-27441-6

**Published:** 2023-01-12

**Authors:** Warda Ahmed, Qaisar Mansoor, Muhammad Sheeraz Ahmad, Tayyaba Zainab, Muhammad Ali Shah

**Affiliations:** 1 University Institute of Biochemistry and Biotechnology (UIBB), Pir Mehr Ali Shah Arid Agriculture University, Rawalpindi, Pakistan; 2 Institute of Biomedical and Genetic Engineering (IBGE), Islamabad, Pakistan; 3 Department of Parasitology and Microbiology, Pir Mehr Ali Shah Arid Agriculture University, Rawalpindi, Pakistan

**Keywords:** Biochemistry, Biological techniques, Cancer, Cell biology, Molecular biology, Plant sciences

## Abstract

*Fagonia cretica* L. is a tropical plant of family Zygophyllaceae with wide range of medicinally important secondary metabolites. The low cellular uptake of the polar compounds in the extract of the plant limits its biological application. In present study efficacy of *F. cretica* modified bioactive nano-formulations for in vitro modulation of TRAIL mediated extrinsic apoptotic pathway as cancer therapy was investigated. *F. cretica* methanolic extracts were formulated at nano-scale for green synthesis of silver nanoparticles, albumin conjugation and liposomes encapsulation to enhance targeted bioactivity against cancer. Physical characterization of the synthesized nanoparticles was done by SEM, EDX and Zeta potential analyzer. In vitro cell viability assay MTT was done for MCF-7, Hep-2, HUH-7 and HCEC cell lines. Relative expression variation of the apoptotic pathway-associated genes was done by qRT-PCR. SEM revealed spherical shape of 56.62 ± 8.04, 143 ± 11.54 and 83.36 ± 38.73 nm size and zeta potential − 18.6, − 15.5 and − 18.3 mV for liposomes, silver and albumin nanoparticles. Silver nanoparticles showed highest anticancer activity in vitro than albumin and liposomes nanoparticles with IC_50_ 0.101 ± 0.004, 0.177 ± 0.03 and 0.434 ± 0.022 mg/mL in MCF-7, Hep-2 and HUH-7 respectively. *F. cretica* albumin and silver nanoparticles upregulated the in vitro TRAIL, DR4, DR5 and FADD gene expression at statistically significant levels in Hep-2 cell lines. Nano-formulations of *F. cretica* proved therapeutically important biomolecules in vitro. The hypothesized modulation of extrinsic apoptosis pathway genes through the plant nanoparticles proved novel medicinal options for effective treatment of cancer and enhancing the bioavailability of the active plant metabolites.

## Introduction

Cancer has been considered a health related concern throughout history accounting for more than nine million fatalities in 2018 and ten million in 2020 due to its high morbidity and mortality^1,2^. There have been numerous attempts to discover natural products that can inhibit the growth and proliferation of cancerous cells, without harming normal and hematopoietic cells at the same time. Anticancer and antibiotic activity shown by medicinal plants have been proven to inhibit the growth and proliferation of cancer cells while reducing chemotherapeutic drug dosage and ameliorating their inherent and severe side effects^3^. Several compounds derived from medicinal plants are used as anticancer drug, e.g. vinblastine, vincristine, paclitaxel, taxol, and phodophyllotoxins^4^.

*Fagonia* *cretica* L. is a tropical plant grows on calcareous rocks in the Mediterranean region of Africa, Pakistan, and Afghanistan. It is commonly called ‘Dhamasa’ in entire Asia. This plant has been found to possess anticancer, antibacterial and anti-inflammatory properties. In addition, in vitro studies indicate that plant has shown non-toxic effect to normal mammalian cells^5^. Moreover, this plant has been used for curing various illnesses including hematological, neurological, endocrine, inflammatory disorders, skin diseases, small pox and endothermic reactions in the body and removal of toxins from body. This range of medicinal importance of the plant is due to the presence of wide range of antioxidants, phenolics, flavonoids and triterpenoids^6,7^.

The use of whole plant extracts originates from nature's wisdom to target one or more cellular signaling pathways to prevent the spread of multiple drug resistance caused by the heterogeneity of cancer cells^8,9^. Plants polyphenols being major antioxidant and anticancer agent works to treat various inflammatory diseases^10–12^. The in vivo action of polyphenol is generally limited by their low stability, bioavailability, and cellular absorption^13,14^. Aqueous extracts of *Fagonia cretica* L. have been reported to induce cell cycle arrest and apoptosis in vitro. The mechanistic involvement of p53-dependent and independent pathways and activation of the DNA damage response were identified for cell proliferation inhibition and natural cell death^15^.

*TRAIL* (tumor necrosis factor (*TNF*)-related apoptosis inducing ligand) is a TNF receptor superfamily homotrimeric type 2 transmembrane protein ligand. A caspase-dependent apoptosis signal transduction pathway initiates extrinsic apoptosis pathway by binding to death receptor 4 (*DR4*) and death receptor 5 (*DR5*) expressed in most cancer cells, but not in normal cells. *TRAIL* is regarded as an anticancer target with little or no toxic effects to normal cells^16–18^.

An interdisciplinary approach between nanotechnology and pharmacognosy help to improve therapeutic window. Improved cellular absorption of drugs can be achieved using nano encapsulation-based medication. Encapsulated natural product extracts could overcome the obstacles faced by polyphenols by improving bioavailability of the active components and allowing passage through cell membranes^19^. Albumin-based drug delivery systems represent a smart approach in which a notable amount of drug can be combined into the particle matrix due to the various drug binding sites present in the albumin molecule^20–22^. It has ability to transport several endogenous or exogenous compounds including fatty acids throughout the whole body. The delivery of anticancer drugs to tumor using albumin NPs has proven to be highly effective^23,24^. Liposomes have also been reported as an effective drug delivery vehicle^25^. The characteristic bilayers of phospholipids make liposomes which can encapsulate hydrophilic as well as hydrophobic compounds^26^. It helps to prevent entrapped molecule degradation and allow controlled release of target molecules^27^. Silver has been recognized for its antimicrobial properties against bacteria found in medical and industrial processes. Green synthesis of silver nanoparticles (NPs) out performs chemical and physical methods in terms of cost, environmental friendliness, and ease of scaling up for large-scale synthesis^28^.

The present study is designed for efficient delivery of *F. cretica* L. plant extract based albumin, liposomes and silver nanoparticles for in vitro anticancer potential against MCF-7, Hep-2 and HUH-7 cancer cell lines along with the plant nanoparticles based treatment dependent transcriptional level expression modulation of apoptotic pathway associated genes TRAIL, DR4, DR5, FADD and TP53 as natural cell death mechanism induction therapeutic option for cancer.

## Materials and methods

### Plant collection

*Fagonia cretica*, belonging to peripheral northern region of Pakistan, was collected and used in the present study as per guidelines and permission approved by the Department of Botany, PMAS-Arid Agriculture University Rawalpindi (PMAS-AAUR), Pakistan. Dr. Rahmatullah Qureshi, a renowned botanist at the university identified the plant. A voucher specimen of the plant (PMAS-06) has been deposited in the public herbarium at PMAS-AAUR, a public sector institution. Dr. Rahmatullah Qureshi (PhD), a renowned botanist at the university identified the plant.

The decomposed, senescence parts and soil particles were removed from the plant. The plant material was washed and dried under the shade. The dried plant was ground to a fine powder of 20 microns.

### Extract preparation

#### Methanolic extract preparation

Powdered ground plant sample 33.33 g was dissolved in 100 mL methanol according to the maceration method. The filtrate was then subjected to rotary evaporator to get the final concentration of the extract^29^. The percentage yield of the extract was calculated by using formula:

$$Yield (\mathrm{\%})=\frac{\mathrm{Dry \, weight \, of \, extract}}{\mathrm{Dry \, weight \, of \, plant \, material }} \times 100$$

#### Aqueous extract preparation

The powdered plant sample of 2 g was soaked in 20 mL distilled water and heated on hot plate for 15 min. The extract was filtered using whatman filter paper and stored at 4 °C.

### Synthesis of silver nanoparticles

The aqueous solution of 80 mL of 1 mM AgNO_3_ containing 20 mL of filtered plant extract was boiled until color changed to dark brown to form Ag^+^ ions. Following this, the solution containing silver nanoparticles was centrifuged at 15,000 rpm for 15 min. The pellet was collected, dried and store at 4 °C^30^.

### Preparation of albumin nanoparticles

Plant based albumin nanoparticles were prepared by simple desolvation method as milky white suspension^31^. 2 mL deionized water was added to 40 mg bovine serum albumin (BSA) and stirred vigorously for 15 min at room temperature. 3 mL plant solution (4 mg extract in 1 mL methanol) was added drop-wise to the above solution at a rate of 1 mL/min under constant stirring at room temperature in dark. In order to stabilize and cross-link the BSA NPs, 20 µL of 25% glutraldehyde was added to the above solution. The stirring was done for 2 h to ensure crosslinking of amino acid residues. After centrifugation at 18,000 rpm for 15 min, the remaining loaded BSA NPs were washed with deionized water, vacuum dried and stored 4 °C.

### Preparation of nano-liposomes from plant extracts

Plant loaded liposomes were made by thin film hydration method^32^. Briefly lipid phase containing egg phosphatidyl choline, PEG2000DSPE, and cholesterol in the ratio of 120:10:20 (mg) respectively were dissolved in 5 mL of chloroform in 100 mL round bottom flask. 10 mg plant extract was mixed with lipid phase and dried using rotary evaporator to form a thin film around walls of the flask. An aqueous phase containing phosphate buffer saline (PBS) buffer pH 7.4 with 3% v/v Tween-80 was added to the lipid phase to prepare a homogenized liposome suspension using a rotary evaporator. The hydrated liposomes were exposed to ultra sonication for three cycles of 15 min each with 5-min rest interval between each cycle, to reduce particle size. The un-entrapped plant extract was removed from the liposomal suspension by centrifugation at 1000 rpm. Lipid pellets were separated, rinsed with the PBS buffer and lyophilized. The dried liposomes were collected and stored at 4 °C until further use.

### Characterization of synthesized nanoparticles

#### Scanning electron microscopy (SEM) and energy-dispersive X-ray (EDX)

The morphology and elemental characterization of NPs was done by SEM (TESCAN VEGA 3) and EDX. The NPs were silver coated using sputter coater for uninterrupted SEM imaging. The EDX application of SEM was used to determine the elemental composition of the nanoparticles.

#### Zeta potential

Zeta potential measurements were performed using the Malvern Zetasizer (Ver 7.12). The NPs suspension of 1 mL was diluted ten times and Zeta potential in mV was measured per instrument guidelines.

### Entrapment efficiency (EE) percentage

The percentage entrapment efficiency of NPs was determined by spectrophotometric analysis^32^ using Nano Drop 2000C (Thermo Scientific, USA). The amount of encapsulated plant extract was calculated by taking the difference in extract present in the supernatant after centrifugation from the amount of extract initially added. The amount of encapsulated plant extract was determined in each freshly prepared albumin, silver and liposomes NPs. The sample was centrifuged at 14,000 rpm for 25 min. Absorbance at 272 nm was measured to calculate the amount of un-encapsulated plant extract after the separation of nano particles pellet (encapsulated extract) by re-centrifuging at 14,000 rpm for 15 min. The following formula was used to determine the amount of drug encapsulated:

$$EE (\mathrm{\%})=\frac{\mathrm{Abs}.\mathrm{ of \, extract \, in \, NP \, suspension }-\mathrm{Abs}.\mathrm{ of \, extract \, in \, supernatant }}{\mathrm{Abs}.\mathrm{ of \, extract \, in \, NP \, suspension }} \times 100$$

### Determination of in vitro antioxidant capacity

DPPH (2,2-diphenyl-1-picrylhydrazyl) radical scavenging assay was used to determined antioxidant potential of plant extract and their NPs using quercetin as standard^33^. The dose response curve was used to calculate IC_50_ values.

### Cell viability and anticancer assay

MCF-7, Hep-2, HUH-7 and HCEC cells were revived in Dulbecco's Modified Eagle's medium (DMEM) supplemented with 10% fetal bovine serum (FBS) and 1% PenStrep/Amphotericin in 75 cm^2^ flask. Cells were incubated at 37 °C with 5% CO_2_ in a humidified incubator to reach 80% confluence.

MTT assay using Thiazolyl Blue Tetrazolium Bromide (MTT Powder) (Sigma-Aldrich, USA) was performed to evaluate the in vitro cell viability and anticancer activity. Briefly, 1 × 10^5^ cells of MCF-7, Hep-2, HUH-7 and HCEC cells were transferred to 96-well tissue culture plate. The cells were treated with defined concentration of 200, 400 and 800 µg/mL of plant extract and NPs. The plate was incubated at 37 °C with 5% CO_2_ for 24 h in water jacketed incubator (Shel Lab, USA). 0.75 mg/mL MTT was added in each well followed by incubation at 37 °C for 3–4 h. 50 µL of Solubilization solution (40% dimethylformamide in 2% glacial acetic acid with 16% sodium dodecyl sulfate) was added to each well and incubated for 30 min in dark at room temperature to dissolve the formazan crystals. Absorbance of the treated and non-treated cell cultures were measured at 570 nm using FLUOstar Omega Microplate Reader (BMG LabTech Australia). Cell viability of cells was calculated by the following equation:

$$\mathrm{Cell \, viability} (\%)=\frac{\mathrm{Absorbance \, of \, sample }(\mathrm{treated \, cells})}{\mathrm{Absorbance \, of \, control }(\mathrm{untreated \, cells})} X 100$$

$$\mathrm{Cell \, Cytotoxicity} \left(\%\right)= 100-\mathrm{Cell \, Viability}\left(\mathrm{\%}\right)$$

IC_50_ was calculated from the dose–response curve of each sample.

### Relative gene expression profiling

An in vitro relative gene expression profiling for TRAIL, DR4, DR5, FADD, c/FLAR and TP53 genes was done at transcriptional level by qRT-PCR using gene specific primers shown in Supplementary Table 1. Hep-2 cancer cell lines as an in vitro model was treated with the IC_50_ concentration of the extract and all the NPs in six well cell culture plate and incubated for 24 h at 37 °C in 5% CO_2_ water jacketed incubator. RNA was isolated from treated and non-treated (control) Hep-2 cells lines using Thermo Scientific GeneJET RNA Purification Kit (Thermo Scientific, USA). cDNA from the extracted RNA samples was synthesized using RevertAid™ first strand cDNA Synthesis Kit (Thermo Scientific, USA). RT-PCR was done by 1 µL of 10 ng cDNA using 1X SYBR Green PCR reagent (Thermo Scientific, USA) with 1 mM forward and reverse primers in SLAN-96P RT-PCR machine (Sansure, China). The two step thermal profile for RT-PCR consisted of incubation at 50 °C for 2 min and initial denaturation at 95 °C for 10 min followed by 45 cycles of 95 °C for 15 s, annealing and data collection at 58 °C for 40 s. *GAPDH* gene was amplified as a housekeeping gene for relative quantification reference for the targeted genes. Relative gene expression for fold change in quantity was determined by ΔΔCt values calculations.

### Statistical analysis

All tests were performed in triplicate. Graphpad prism version 9.0 was used to perform statistical analysis on data generated from replicates of the experiments. The values are reported as mean ± standard deviation. One way ANOVA was used to evaluate the data followed by Dunnett's multiple comparison tests. A *p*-value < 0.05 was considered as significant.

## Result and discussion

### Extraction yield

Extraction of 33.33 g of a finely powdered sample of the *F. cretica L.* whole plant led to a 19% extraction yield which is 6.33 g from the methanolic solvent.

### Characterization of NPs

#### SEM

**Figure 1:**
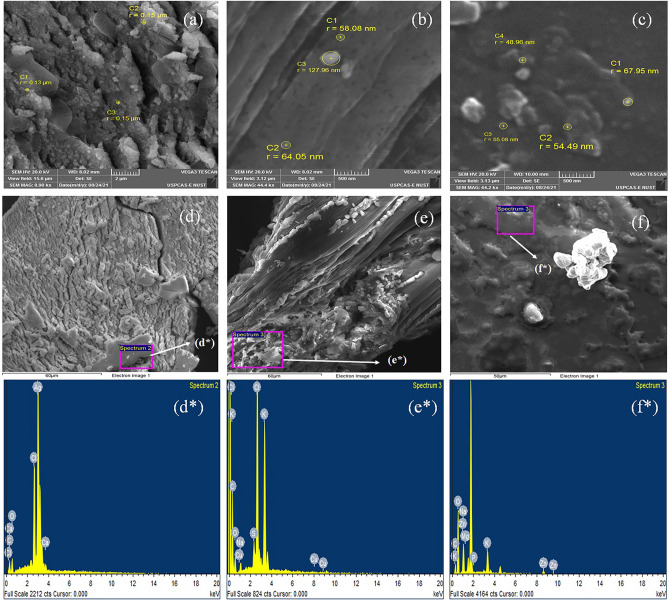
SEM images of *F. cretica* (**a**) Silver NPs (**b**) Albumin NPs (**c**) Liposomes NPs with size indication of the NPs. Mapping of the NPs for EDX (**d**) Silver NPs (**e**) Albumin NPs (**f**) Liposomes NPs. (d*) Silver NPs (e*) Albumin NPs (f*) Liposomes NPs EDX elemental output. (d*–f*) regions are marked in the (**d**–**f**).

The SEM images of *F. Cretica L* NPs of silver, albumin and liposomes revealed the uniformity of spherical-shape with a size range of 143 ± 11.54, 83.36 ± 38.73 and 56.62 ± 8.04 nm respectively (Table 1). The SEM images are shown in Fig. 1.

**Table 1:** Characterization of NPs from *F. cretica* plant extract.

Sample	Size (nm)	EE (%)	Zeta potential (mV)
Liposomes NP	56.62 ± 8.04	72.53 ± 2.914	− 18.6
Silver NP	143 ± 11.54	64.16 ± 2.538	− 15.5
Albumin NP	83.36 ± 38.73	70.86 ± 1.457	− 18.3

#### EDX spectroscopic analysis of NPs

Strong atomic signals of Ag, C and O atom with intensity of 62.16, 75.6 and 80.81% in silver, albumin and liposomes nanoparticles were identified respectively. The atomic spectrums from all the NPs are shown in Fig. 1.

#### Zeta potential

**Figure 2:**
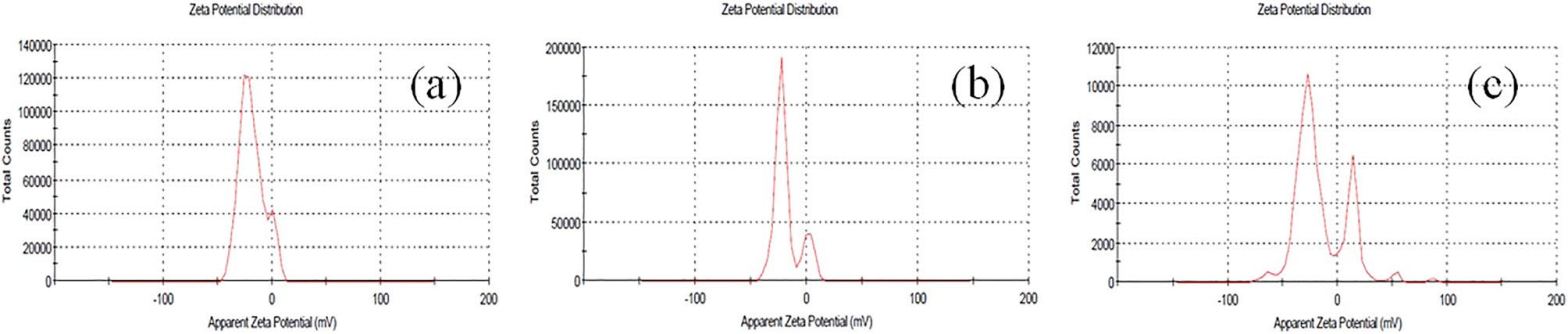
Zeta potential of F. cretica NPs (**a**) liposomes NP (**b**) Silver NP (**c**) Albumin NP.

Liposomes, silver and albumin NPs showed zeta potential − 18.6, − 15.5 and − 18.3 mV respectively as shown in Fig. 2 which indicates good stability of the prepared NPs (Table 1).

### Entrapment efficiency (EE)

The EE measurements provided 72.53 ± 2.914, 64.16 ± 2.538 and 70.86 ± 1.457% encapsulation efficiency for liposomes, silver and albumin NPs respectively (Table 1).

### Antioxidant assay

**Figure 3:**
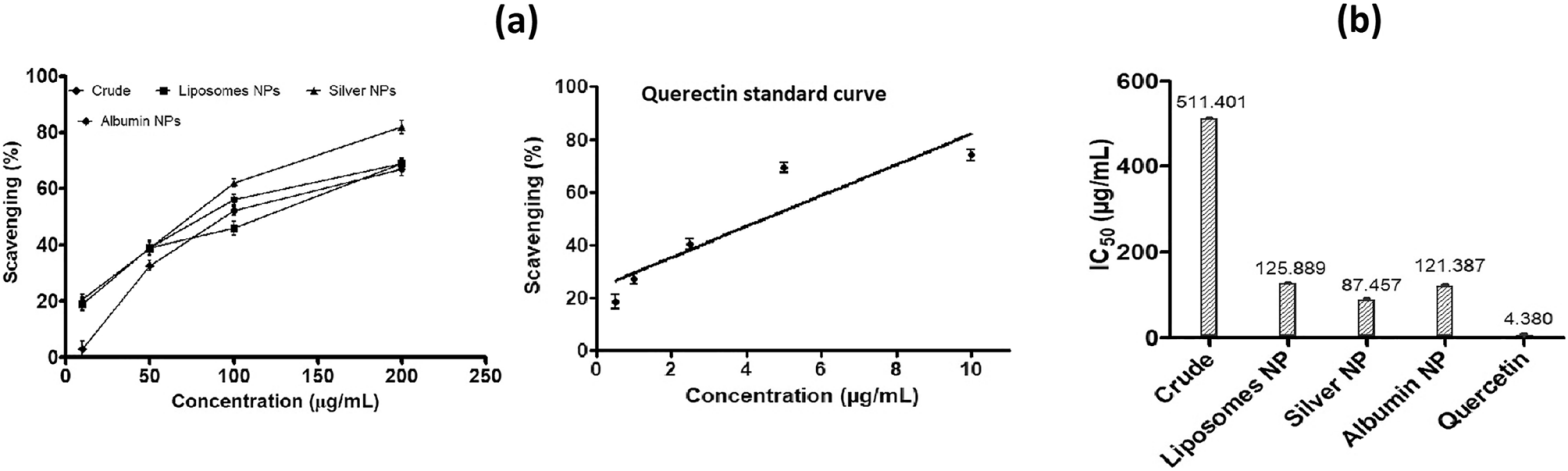
DPPH Assay (**a**) % scavenging effect of plant crude extract and their NPs (**b**) IC_50_ of *F. cretica* plant extract and its NPs.

The percentage scavenging by DPPH assay using 10, 50, 100 and 200 µg/mL concentrations of crude plant extract and the NPs showed dose-dependent increase in antioxidant activity. Silver NPs showed highest scavenging in terms of percentage with IC_50_ value of 87.5 ± 2.52 µg/mL. Albumin and Liposomes NPs have IC_50_ value 121.39 ± 3.19 and 125.89 ± 1.73 µg/mL respectively as shown in Fig. 3. NPs of silver, albumin and liposomes showed enhanced antioxidant activity as compared to the crude plant extract, IC_50_ 511 ± 4.03 µg/mL.

### Cell viability and anticancer activity

**Figure 4:**
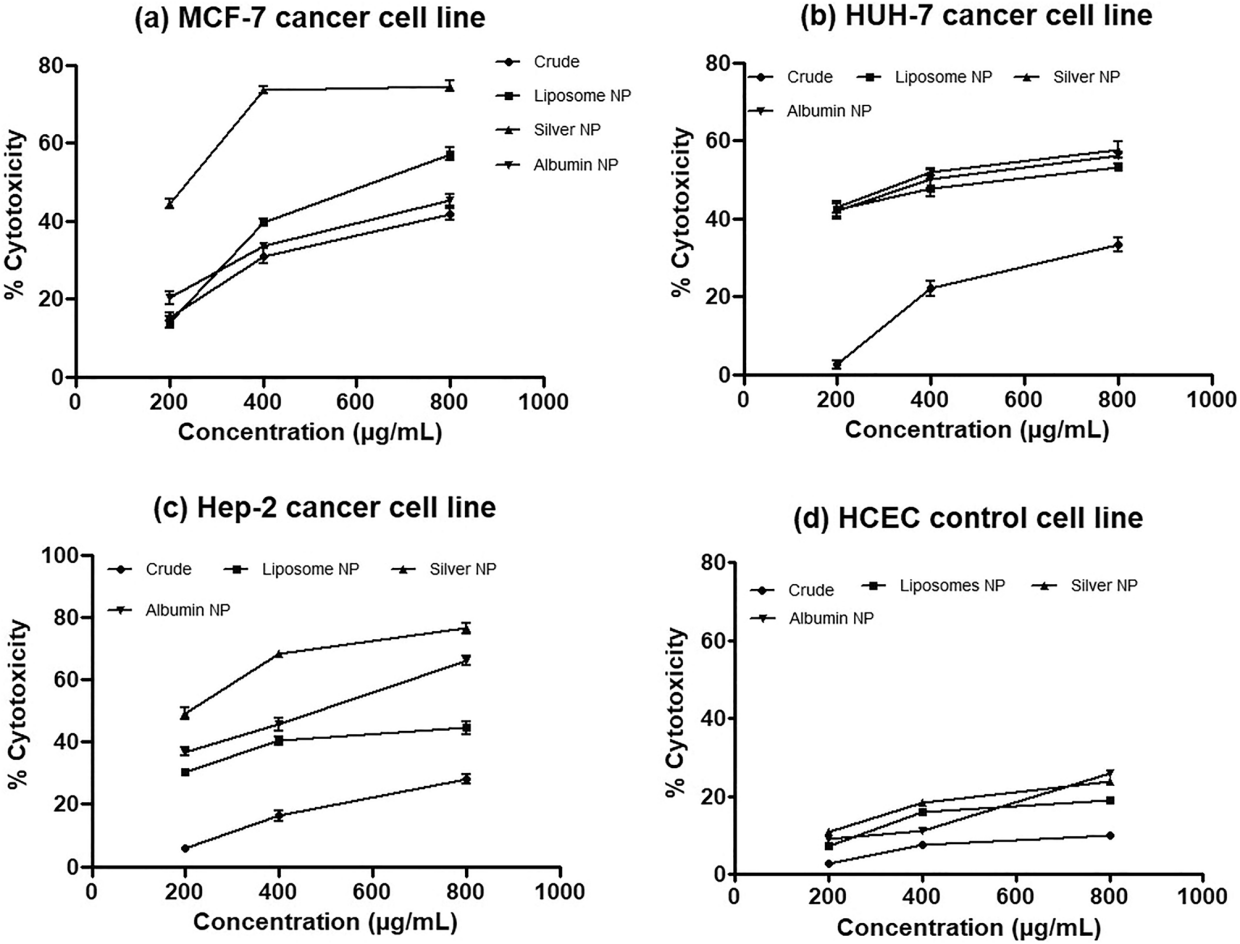
Anti cancer activity (detemined by MTT cell viability assay) of *F. cretica* plant extract and their NPs against (**a**) MCF-7 (**b**) HUH-7 (**c**) Hep-2 and (**d**) HCEC cell lines. Data represented as mean ± SD of three independent experiment.

**Figure 5:**
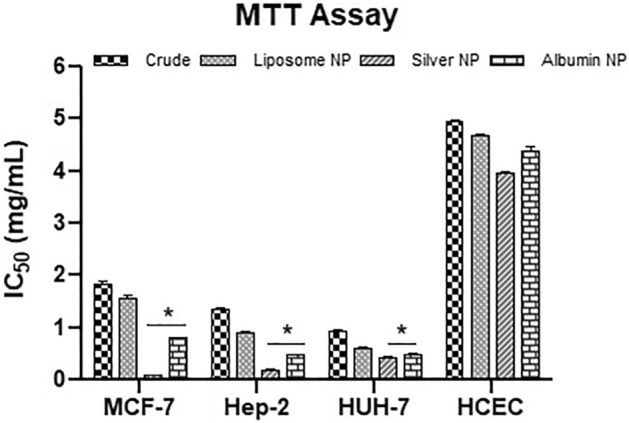
IC_50_ of *F. cretica* plant extract and their NPs against MCF-7, Hep-2 and HUH-7 cell lines. Data denoted *(p < 0.05) are significant compared to control.

*Fagonia cretica* extract and the NPs showed cytotoxicity in dose-dependent manner as shown in Fig. 4. Silver NPs showed significant (0.0219) anticancer activity (76%) with IC_50_ of 0.1 ± 0.004, 0.17 ± 0.04 and 0.4 ± 0.022 mg/mL while the albumin NPs with IC_50_ of 0.8 ± 0.005, 0.47 ± 0.01 and 0.49 ± 0.04 mg/mL showed anticancer potential against MCF-7, Hep-2 and HUH-7 cancer cell lines respectively (Fig. 5) and the liposomes NPs showed anticancer activity with IC_50_ 1.5 ± 0.04, 0.9 ± 0.05 and 0.59 ± 0.04 mg/mL against the cancer cell lines respectively. Interestingly, all the synthesized NPs showed remarkably higher anticancer activity than crude plant extract against cancer cell lines. No notable cytotoxicity of the extract and the nanoparticles was observed in normal cell line HCEC for concentration as high as 800 mg/mL.

### Relative gene expression profiling RT-PCR

**Figure 6:**
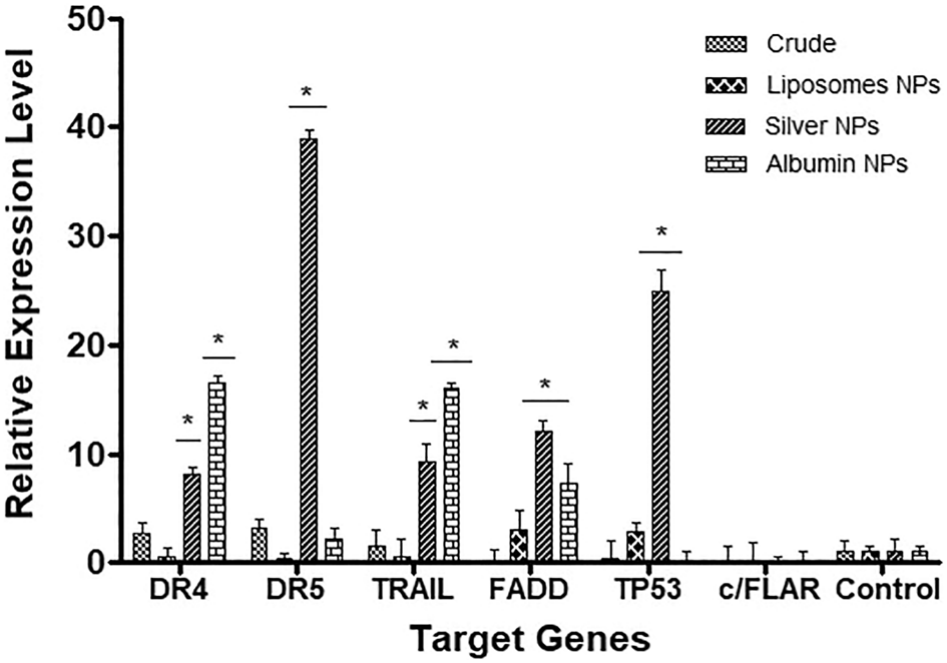
qRT-PCR based relative expression levels of the genes involved in TRAIL mediated apoptosis after treatment of the Hep-2 cancer cell lines with *F. cretica* plant extract and their NPs. Data denoted *(p < 0.05) are significant compared to control analyzed by one-way ANOVA with Dunnett’s multiple comparison post-test.

The impact of *F. cretica L* plant extract and its NPs formulations i.e. silver, albumin and liposomes on the post transcriptional expression level of TRAIL, DR4, DR5, FADD, cFLAR and TP53 was determined. Silver and albumin NPs showed statistically significant increase in relative expression of genes as compared to crude plant extract with p-value 0.0150 and 0.0343 respectively. Silver and albumin NPs relative increase in expression of DR4 gene accounting for up to 16.4 and 8.1 folds rise respectively. Silver NPs accordingly showed a boosted rise, 38.3 folds in the relative expression of DR5 gene whereas albumin and liposomes did not show any impact on the gene expression. A 15.9 and 9.3 fold increase in TRAIL gene expression was observed as response for albumin and silver NPs treatment. Notably again the FADD gene was upregulated to 12.1 folds by silver NPs as compared to 7.2 fold increase by the albumin while TP53 gene was upregulated to 24.9 folds by silver NPs. Surprisingly no changes in expression of cFLAR was noted in either case. Results are shown in Fig. 6.

## Discussions

Cancer is a multifaceted disease and the available therapeutic options are either limited in their efficacy or involved in the triggering the undesirable side effects and even morbid complications while targeting the cancer. Natural plant products are real hope as a matter of their less toxic, safe and target oriented gene regulation at cell signaling level. Biocompatible adaptations of plant metabolites are one of the promising techniques to concentrate the modest and effectual content concentration at the site of action. Taking advantage of the flexible modification options at nano-scale level *F. Cretica* L. extracts formulations of silver, albumin and liposomes were prepared and tested for antioxidant activity, anticancer potential and effective targeting of the extrinsic apoptotic pathway associated genes.

The nano sized spherically shaped *F. cretica* made sliver; albumin coupled, and liposome encapsulated NPs showed 50 to 80 nm diameter through SEM. Strong atomic signals of Ag in silver NPs, C and O for albumin and liposomes a predictive of competent entrapment efficiency, were observed in EDX. These characterizations are well comparable with the nano formulations reported earlier^31,32,34^. The NPs with these characteristics indicate adequate basis to be tested as therapeutic preference against cancer cells.

The antioxidant potential of the *F. cretica* NPs observed for silver with IC_50_ value of 87.5 ± 2.52, albumin 121.39 ± 3.19 and liposomes 125.89 ± 1.73 µg/mL revealed strong free radical scavenging hallmark with fine nano tunable modifications^35^.

Complementing the exuberant antioxidant inbuilt properties, the NPs showed strong anticancer array in cancer cell lines in vitro*.* Decisively the non-cytotoxic effect of the NPs on normal cell lines comprehends their bio efficiency and bio safety. MCF-7, Hep-2 and HUH-7 as in vitro model of pre-clinical trials for cancer research used in this study showed strong sensitivity against all the NPs synthesized as prospective treatment choice. Previously the green synthesized silver NPs using aqueous extract of *F. cretica* showed enhanced in vitro anticancer activity in breast cancer cell line^15^. Similarly Poly (d, l, lactic-co-glycolic acid)-poly (ethylene glycol) NPs loaded with pomegranate extract showed enhanced in vitro bio efficiency against breast cancer line^36^. Moreover the smart usefulness of Curcumin loaded bovine serum albumin loaded NPs in vitro showed strong anticancer activity^37^. The manifold levels of anticancer activity of the *F. cretica* NPs in preclinical in vitro models for the present work are pertinently reported here for the first time with improved cancer cells growth proliferation inhibition in vitro*.*

Insights into the modest mechanism for a compelling cancer cell growth arrest by the plant NPs, the extrinsic apoptotic pathway player TRAIL, DR4, DR5, FADD, TP53 and cFLAR genes were evaluated as molecular therapeutic targets by these NPs. *F. cretica* silver and albumin NPs showed a statistically significant increase in the expression of TRAIL, DR4 and FADD, while the liposome NPs did not showed such a striking augmentation in fueling the target genes expression. Silver NPs also showed bloom in the transcriptional level relative gene expression of DR5. Interestingly c/FLAR gene remained under-expressed in either of the case thus an uninterrupted TRAIL gene increased expression can be regarded in vitro*.* These new and latest findings from the study are suggestive of strong evidence of natural products efficiency in phenomenal modulation of apoptosis associated genes to treat cancer. The apoptosis inducer TRAIL molecule through interaction with the death receptors DR4 and DR5, all three are important biomedical targets for cancer treatment and undergoing clinical trials in various stages^38^. The new and latest findings from the present study are suggestive of strong evidence of nano-scale modified *F. cretica* extracts as efficient therapeutic choices for preclinical and in vivo trials for phenomenal modulation of apoptosis associated genes, taking the likelihood concept to cancer treatment.

## Conclusions

The inquisitive quest for natural product nano models payload at the target sites harvested in the present research objectively proved the enhanced bioavailability and bioactivity of *F. cretica* NPs. The plant formulated silver and albumin coupled NPs narrated the absolute composition as in vitro anticancer moieties and revitalizing the expression of abandoned and forgotten apoptotic pathway associated genes TRAIL, dependent or independent of TP53. This class of *F. cretica* NPs has been discovered and presented for the first time to be showcased in the oriental cancer therapy and systems medicine.

## Supplementary Information

Supplementary Table 1.

## Data Availability

All the data generated and/or analyzed during this study are included in this research article and its supplementary information files.
