# Supplementary material for: TRAIL mediated apoptosis ruling and anticancer trigger by fine-tuned nano spheres of Fagonia cretica methanolic extracts as novel cancer regime
Source: Sci Rep. 2023 Jan 12;13:671. doi: 10.1038/s41598-023-27441-6 (PMC9837038; doi:10.1038/s41598-023-27441-6)
Supplement: Supplementary file 1 — Supplementary Table 1. [file 41598_2023_27441_MOESM1_ESM.docx]

Supplementary Table 1: Sequence of primer used for determined relative expression of the genes involved in TRAIL mediated apoptosis by qRT-PCR

| **S. No.** | **Genes** | **Sequences (5’-3’)** |
| --- | --- | --- |
| 1. | DR4-Forward | AGAGAGAAGTCCCTGCACCA |
|  | DR4-Reverse | GTCACTCCAGGGCGTACAAT |
| 2. | DR5- Forward | TGCAGCCGTAGTCTTGATTG |
|  | DR5- Reverse | TCCTGGACTTCCATTTCCTG |
| 3. | TRAIL- Forward | GGAACCCAAGGTGGGTAGAT |
|  | TRAIL- Reverse | TCTCACCACACTGCAACCTC |
| 4. | FADD- Forward | CCGAGCTCAAGTTCCTATGC |
|  | FADD- Reverse | CGTTAAATGCTGCACACAGG |
| 5. | c/FLAR- Forward | TGATGGCAGAGATTGGTGAG |
|  | c/FLAR- Reverse | TCTGGGGCAACCAGATTTAG |
| 6. | TP53-Forward | GGCCCACTTCACCGTACTAA |
|  | TP53- Reverse | GTGGTTTCAAGGCCAGATGT |
| 7. | GAPDH- Forward | ACCACAGTCCATGCCATCAC |
|  | GAPDH- Reverse | TCCACCACCCTGTTGCTGTA |
